# Estimating the potential for global dissemination of pandemic pathogens using the global airline network and healthcare development indices

**DOI:** 10.1038/s41598-022-06932-y

**Published:** 2022-02-23

**Authors:** Margaux M. I. Meslé, Roberto Vivancos, Ian M. Hall, Robert M. Christley, Steve Leach, Jonathan M. Read

**Affiliations:** 1 National Institute for Health Research, Health Protection Research Unit in Emerging and Zoonotic Infections at University of Liverpool, Institute of Infection and Global Health, The University of Liverpool, Waterhouse Building (2nd Floor, Block F), 1-5 Brownlow Street, Liverpool, L69 3GL UK; 2 Institute of Infection and Global Health, The University of Liverpool, 8 West Derby Street, Liverpool, L69 7BE UK; 3 Field Service, National Infection Service, Public Health England, Suite 3B Cunard Building, Water Street, Liverpool, L3 1DS UK; 4 National Institute for Health Research, Health Protection Research Unit in Gastro Intestinal Infections at University of Liverpool, Institute of Infection and Global Health, The University of Liverpool, Waterhouse Building (2nd Floor, Block F), 1-5 Brownlow Street, Liverpool, L69 3GL UK; 5 Department of Mathematics, Alan Turing Building, The University of Manchester, Manchester, M13 9PL UK; 6 Emergency Response Department, Health Protection Directorate, Public Health England, Porton Down, Salisbury, SP4 0JG Wiltshire UK; 7 National Institute for Health Research, Health Protection Research Unit in Emergency Preparedness and Response at Kings College London, Department of Psychological Medicine, King’s College London, Weston Education Centre, Cutcombe Road, London, SE5 9RJ UK; 8 National Institute for Health Research, Health Protection Research Unit in Modelling Methodology at Imperial College London, Level 2, Faculty Building, South Kensington Campus, London, SW7 2AZ UK; 9 Lancaster Medical School, Lancaster University, Lancaster, LA1 4YG UK; 10 Present Address: World Health Organization (WHO) Regional Office for Europe, Copenhagen, Denmark

**Keywords:** Infectious diseases, Health policy, Epidemiology

## Abstract

Pandemics have the potential to incur significant health and economic impacts, and can reach a large number of countries from their origin within weeks. Early identification and containment of a newly emerged pandemic within the source country is key for minimising global impact. To identify a country’s potential to control and contain a pathogen with pandemic potential, we compared the quality of a country’s healthcare system against its global airline connectivity. Healthcare development was determined using three multi-factorial indices, while detailed airline passenger data was used to identify the global connectivity of all countries. Proximities of countries to a putative ‘Worst Case Scenario’ (extreme high-connectivity and low-healthcare development) were calculated. We found a positive relationship between a country’s connectivity and healthcare metrics. We also identified countries that potentially pose the greatest risk for pandemic dissemination, notably Dominican Republic, India and Pakistan. China and Mexico, both sources of recent influenza and coronavirus pandemics were also identified as among the highest risk countries. Collectively, lower-middle and upper-middle income countries represented the greatest risk, while high income countries represented the lowest risk. Our analysis represents an alternative approach to identify countries where increased within-country disease surveillance and pandemic preparedness may benefit global health.

## Introduction

The emergence of pandemic influenza in Mexico in 2009, the outbreak of Zika virus in the Americas in 2015, and the emergence of the SARS-CoV-2 in China in 2019 highlight the need for an effective response when pathogens with potential for large outbreaks emerge. Early detection and containment at source are crucial in reducing morbidity and mortality in the source country, and for the global community to prevent a potential pandemic^1^. Countries with established surveillance mechanisms, an organised healthcare system with appropriate facilities, equipment and trained staff are more likely to rapidly detect and control an outbreak than countries without such provisions^2,3^. Understanding the global resilience of healthcare systems to pandemic and outbreak emergence events is, therefore, a crucial step in allocating resources to improve surveillance and public health interventions^4^. In 2016, the cost of a pandemic was estimated at $60 billion per year, greatly outweighing the cost of improving country preparedness (estimated at $4.5 billion per year), and only representing a fraction of the spending for financial crises prevention^5^. It has been estimated in 2021 that the SARS-CoV-2 pandemic may generate global economic losses of up to $8.8 trillion^6^. While access to quality healthcare has improved recently in many countries, the global disparity of healthcare access is widening^7^.

Accurate and timely surveillance systems increase the opportunity for early outbreak detection^1^. In 2005, the World Health Organization (WHO) updated its International Health Regulations (IHR), which requires member nation states to report outbreaks posing a potential threat to the international community in a timely manner^1^. More recently (in 2018), the WHO and World Bank co-created the Global Preparedness Monitoring Board, aiming to understand and address country resilience towards outbreaks, by holding stakeholders accountable and ensuring outbreak preparedness is kept a priority in member state agendas^8^. The WHO also requests that member states voluntarily undergo a Joint External Evaluation (JEE) to determine their capacity to respond to a public health event^8^.

The international spread of infectious diseases of humans is driven by the movement of infected individuals^9^. The global airline network is expanding, linking geographically distant countries by allowing passengers to travel at an increasingly faster pace and in greater numbers^10^. Airline travel is the main mode of access to some remote locations and is the most likely means of disease dissemination over large distances^11^. Although there is an increasing number of flights and passengers travelling as countries are increasingly well connected^12^, some countries—particularly island nations—are still difficult to reach and therefore remain distantly connected within the network^13^. With increasing global connectivity, understanding the implications of a possible pandemic source is beneficial to the global community.

Here, using information from the global airline network and country-specific healthcare indices, we assess the potential for a country to contain an outbreak within its borders and the possible risk to other countries following non-containment and its global connectivity through airline traffic.

## Methods

### Global connectivity measurement

To determine the global connectivity of each country, we developed a network percolation model using global airline passenger data from OAG’s Traffic Analyser database (http://www.oag.com)^14^. Data was extracted between August 2014 and July 2015, and comprised the number of monthly bookings from any origin airport to any destination airport, including direct and indirect routing, and including domestic and international flights^15^. The flight data included connections or layover information, but we did not consider the time that passengers may spend in transit between origin and destination as contributing to a country’s connectivity. Monthly information was extracted from February 2010 to May 2015. This was subsequently aggregated to country level, to generate weighted adjacency matrices of passenger flow based on the number of bookings rather than passenger numbers between countries for each month in the study period. It is important to note that domestic travel (trips originating and terminating within the same country) were excluded from this analysis. However, a multi-legged trip consisting of at least one national and one international leg would be included.

The percolation model simulated the spread of ‘information’ through a network, where nodes (countries) exist in one of two states: empty or occupied. The model is analogous to a Susceptible-Infected (SI) epidemic model (where an ‘infected’ remained so for the duration of the model simulation) but should not be considered equivalent to a pandemic model as no within-country transmission occurs, and the ‘force of infection’ represented by an occupied country remains constant for the duration of the simulation. Within a simulation, the probability that node $i$ with empty status becomes occupied at time $t$ is determined by the number of occupied nodes it is connected to, the rate of passenger flow along the network edges for a specific month ($F$) and a percolation rate coefficient, $\beta$ of value 10^–6^ (an arbitrary value that served to maximise the variation in connectivity measurement between countries). This probability is defined:

$$p_{i} \left( t \right) = 1 - exp\left( {\mathop \sum \limits_{j \in \Psi \left( t \right), j \ne i} F_{ij} } \right)$$

where $j$ denotes other nodes, and ${\Psi }\left( t \right)$ is the set of occupied nodes at time $t$. This model was implemented as a discrete time, stochastic Markov process, where all nodes are empty at the start of a simulation apart from a designated node, the seed country. Simulations proceeded until either at least 25% of all nodes had been occupied at which point the simulation time ($t_{end}$), was recorded, or were timed out at $t = 2000$. The connectivity, $\varphi$, of a country was assumed to be related to the time for the percolation to reach 25% all nodes, and is given by $1/t_{end}$. Note that $t$ does not relate to secular time, and the adjacency matrix, $F$, did not change during the course of a simulation. For each seed country (243 countries in total) and month (64 months in total), 1000 realisations were performed, and the median and 2.5% and 97.5% percentiles of $\varphi$ calculated for each seed country and month combination. The seed country and month with greatest value of $\varphi$ ($\varphi_{max}$) was identified, and used to normalise all values of $\varphi$, ensuring the measure of connectivity was bounded between 0 and 1.

### Estimating healthcare response

Indices quantifying the quality of healthcare systems were used instead of single factors representing various aspects of healthcare systems, such as vaccination, tuberculosis treatment rates, among others. The use of multiple factors was supported by Moore et al*.*^3^ and Hosseini et al*.*^16^ who also found that healthcare systems could not be adequately represented by a single factor. A Spearman correlation test was performed to determine if the index scores correlated with each other. We used three existing, validated indices of measures of national healthcare systems, with publicly available data, as proxies for healthcare provision and the ability of a country to respond and contain an outbreak of a pathogen with large epidemic or pandemic potential (see Table S1). The authors were aware of other indices under development or in process of publication at time of writing, including the WHO endorsed Joint External Evaluation (JEE)^17^, which we excluded as too few countries were included in their analysis. Indices used from all three sources—Rand, Global Health Burden, and Global Health Security—, were normalised such that the lowest scoring country in each had a value of zero, and the highest scoring country had a score of 1. We provide a brief description of each index’s methods here, and refer readers to the source material for full details.

#### Rand index

The Rand’s Infectious Disease Vulnerability Index^3^ was based on 37 factors grouped into seven so-called domains, namely: ‘Health Care’, ‘Public Health’, ‘Economic’, ‘Disease Dynamics’, ‘Political-International’, ‘Political-Domestic’ and ‘Demographics’. The study authors identified these factors following a (non-systematic) literature review. Data was collected from the World Bank and the WHO where available and missing data imputed based on mean GDP and geography of similar countries, before standardising the country scores to range between zero and one. The index authors calculated the average of each domain’s factor score as the final country score, ranging between zero (most vulnerable) and one (most resilient).

#### Global Health Burden (GBD) index

The authors of the Healthcare Access and Quality Index^7^ mapped the Global Burden of Disease (GBD) study to the Nolte and McKee list of amenable burden indicators^18^, retaining 32 of the 33 indicators. This data was available for 195 countries, ranging between the years 1990 and 2015. Some indicators were risk standardised by the authors, using global population levels before calculating a summary measure value for each indicator ranging between zero (low) and 100 (high). The average value of all indicators per country was the final score assigned to each country.

#### Global Health Security (GHS) index

To develop the GHS index^19^, a team of experts identified 140 questions deemed of importance based on a literature review and input from external parties. These questions were classified into six categories (‘Prevention’, ‘Detection and Reporting’, ‘Rapid Response’, ‘Health System’, ‘Compliance with International Norms’ and ‘Risk environment’), from which 34 indicators and 85 sub-indicators were identified. The questions were a mix of quantitative and qualitative, each with a score ranging between zero (lowest) and 100 (highest). The average country score from all 140 questions was used to rank all countries into three tiers: ‘low scores’ (scores up to 33.3), ‘moderate scores’ (scores ranging between 33.4 and 66.6) and ‘high scores’ (scores above 66.7).

Both Rand and GBD indices consider the year 2015, which overlaps with our airline data, but the year for which the GHS index is most aligned was unclear. Combined, the indices considered a total of 206 countries (the number within each index varies). To explore the relationship between connectivity and healthcare indices we performed Spearman correlations (with bootstrapped confidence intervals), and fitted penalized splines (using generalised additive models).

### Gauging a country’s potential for pandemic dissemination

We defined a ‘Worst Case Scenario’ (WCS) as a notional country with the highest connectivity score and the lowest healthcare index score. We assumed this worst case scenario was a country with the highest levels of connectivity ($\varphi = 1$) and a preparedness index score of zero. We assumed connectivity and healthcare scores were weighted equally, and identified each country’s proximity, $d$, to this WCS point as:

$$d_{i} = \sqrt {y_{i}^{2} + \left( {1 - \phi_{i} /\phi_{max} } \right)^{2} }$$

where $\varphi_{i}$ and $y_{i}$ were the connectivity and preparedness index values for country $i$, respectively. To convert the distance measure into a more easily interpretable variable, we calculated $\sqrt 2 - d_{i}$ for each country, where the notional WCS country would score 1 and a country with furthest distance from the WCS point would score 0, to reflect the epidemic risk attributable to the combination of healthcare quality and connectivity. The mean scores for each income group for 2015 (as defined by the World Bank^20^) was also calculated. The World Bank ranked countries in four income groups, namely: low income (n = 34); lower-middle income (n = 48); upper-middle income (n = 55) and high income (n = 59). Only countries for which the distance to WCS could be calculated (which had both a healthcare score and a global connectivity score) were used in analyses; due to country differences between indices, we include all countries in Table S1. Clustering of $d$ values was achieved using a principled k-means algorithm (pamk function in R package fpc^21^) which selects the number of clusters to optimise the average silhouette width. We also categorized countries by their position within quadrants defined by the median connectivity and normalized healthcare indices.

## Results

**Figure 1:**
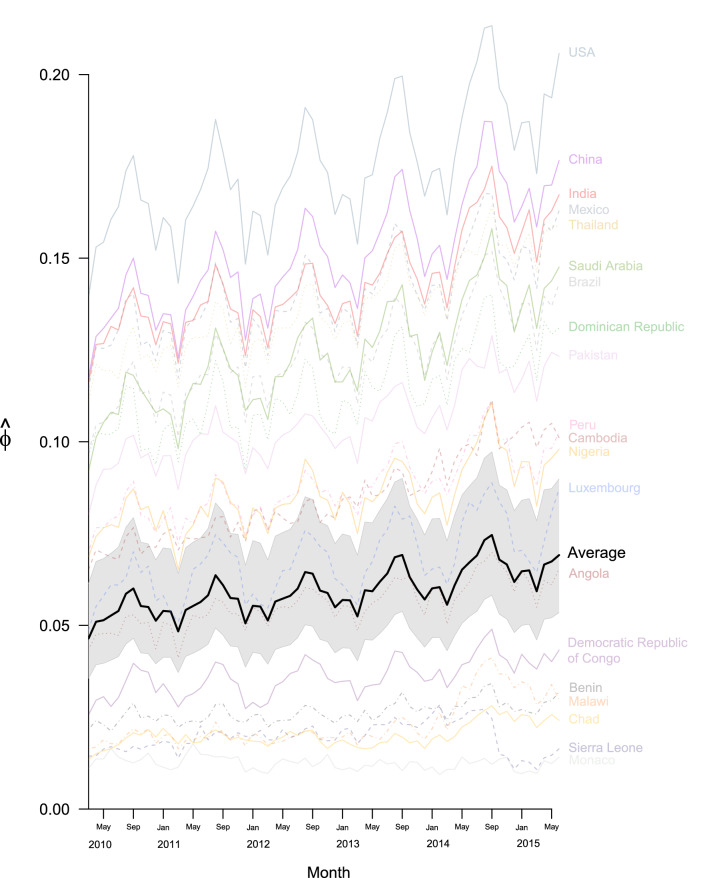
Average global connectivity ($\hat{\upphi }$) for the period ranging from January 2010 to May 2015, highlighting the global average (with confidence intervals in grey) as well as specific countries of interest.

Connectivity of the global airline network has increased over the time period considered (February 2010 to May 2015), with marked seasonal trends, both at the individual country and global levels (Fig. 1). Northern hemisphere summer months (especially July and August) showed the highest connectivity in almost all countries represented, with an additional (although smaller) peak late in the year around December. The United States of America (USA) and China recorded the highest connectivity ($\varphi$) for every month, which were much higher than the global average. The USA saw the highest connectivity for the period considered ($\varphi_{max} = 0.213$) recorded in August 2014, whereas China saw its highest connectivity in July 2014 ($\varphi_{max} = 0.878$). India also had a very good connectivity and increased even more in 2014. Some of the countries in sub-Saharan Africa (such as Benin, Malawi, Chad) were seen to have much lower connectivity compared to the rest of the world, whereas others, such as Nigeria had connectivity levels above the global average. Tuvalu and Kiribati had the lowest connectivity (both ${ }\varphi_{min} = 0$) across all months. Finally, the impact of the West African Ebola outbreak on travel can clearly be seen in Sierra Leone, with the country’s connectivity falling sharply in mid-2014.

Country connectivity broadly corresponds to the income status of that country: the majority (85%) of countries with high connectivity ($\varphi > 0.52$, third quartile for May 2015) classified as high or upper-middle income by the World Bank^20^ (see Table S1), and the majority (68%) of countries with low connectivity ($\varphi < 0.14$, first quartile for May 2015) were classified as low or lower-middle income.

Three independent multifactorial indices were used as proxies to measure a country’s healthcare system and ability to respond to and contain an emerging pandemic: the Rand Corporation’s Infections Disease Vulnerability Index^3^ (hereafter referred to as the Rand Index), the Global Burden of Disease’s Healthcare and Quality Access Index^7^ (hereafter the GBD index), and the Global Health Security Index^19^ (hereafter the GHS index). While Rand and GBD indices were developed for different purposes (GBD to understand the level of healthcare development within each country and Rand to understand the global level of pandemic preparedness) and use different metrics (see respective publications for detailed list of factors), we found that both represented healthcare in a holistic manner and suited our aim of identifying potentially more vulnerable systems. The GHS index was specifically designed to identify country-level capability for detecting and responding to epidemics and emerging pandemics^19^. The scores from these indices are referred to as ‘healthcare index scores’ hereafter.

We found the indices showed good agreement with each other in their country scores (Rand/GBD Spearman correlation coefficient 0.91, 95% CI 0.88–0.93, *p* value < 0.001; Rand/GHS 0.74, 95% CI 0.65–0.80, *p* value < 0.001; GBD/GHS 0.68, 95% CI 0.59–0.76, *p* value < 0.001). Higher normalized index score was associated with a higher country income status for all three indices. The mean score within each income group for Rand was 0.26, 0.44, 0.59 and 0.81 for low, lower-middle, upper-middle- and high income groups, respectively. For GBH the values were 0.36, 0.48, 0.62, and 0.82, while for GHS the values were 0.23, 0.27, 0.33 and 0.54 for the respective income groups. Connectivity generally increased with income group: $\varphi_{mean}$ for low, lower-middle, upper-middle and high income countries was 0.20, 0.29, 0.38 and 0.55 respectively.

**Figure 2:**
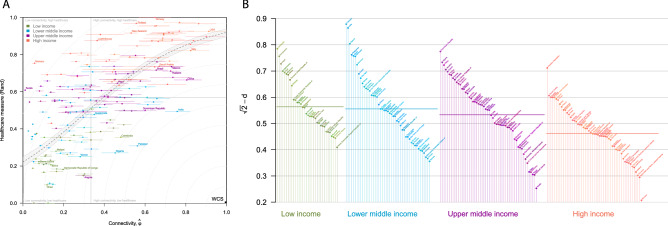
(**A**) Distribution of countries according to their average connectivity ($\hat{\upphi }$) and healthcare development index (Rand index). Each country is colour-coded according to their World Bank income group. The dashed line is a fitted spline with 95% confidence intervals denoted by the grey area. The annotated WCS point represents the ‘Worst Case Scenario’ (greatest connectivity but worst healthcare index), and grey dotted arcs indicate equivalent distance from the WCS. (**B**) Proximity measurement to the Worst Case Scenario (WCS) for all countries, derived using the Rand index. Countries are grouped by World Bank income category and ranked within group. Countries close to the WCS score high, while countries further away from the WCS score low. Group mean averages are denoted by coloured horizontal lines.

Generally, there was a positive relationship between a country’s connectivity and its Rand healthcare index score (Fig. 2A; Figure S3), which was maintained despite seasonal changes in connectivity (Figure S1). We found significant positive correlations between all three normalized healthcare indices and connectivity (Rand 0.67, 95% CI 0.57–0.74, *p* < 0.001; GBD 0.67, 95% CI 0.57–0.74, *p* < 0.001; GHS 0.79, 95% CI 0.72–0.84, *p* < 0.001). Similar patterns and groupings of countries could be seen for all three index scores (Fig. 2; Figure S3; Figure S4; Figure S5; Table S1), where countries classified as low or lower-middle income showed low healthcare scores and low connectivity, and vice versa. However, not all high-income countries had high connectivity (Greenland and Slovenia, for example) and not all low-income countries had low connectivity (Bangladesh and Cambodia, for example), as shown in Table S1.

Some countries were outliers to the general trend. Countries with good connectivity (defined as $\varphi >$ 0.66) but normalized healthcare index scores below the median (0.57 for Rand, 0.60 for GBD, 0.31 for GHS) were India and Mexico. A number of small island nations (such as Tonga and Samoa) had healthcare scores ranging between 0.58 and 0.63 but connectivity scores below 0.60. A number of high income countries, including Iceland and Luxembourg, had high healthcare scores but low connectivity (Table S1), which may be an artefact of focussing our connectivity metric based on commercial air transport while excluding land or sea transportation links. Twelve countries (Canada, France, Germany, Italy, Japan, Netherlands, Spain, South Korea, Switzerland, Thailand, United Kingdom, USA) had high connectivity (upper decile $\varphi$) and high healthcare scores (upper decile).

We identified a number of countries with closest proximity ($d$) to the notional Worst Case Scenario (WCS) which represents a country with poor healthcare and high global connectivity. Cluster analysis of $d$ values based on the Rand healthcare index identified 11 countries as having the smallest $d$ values: Algeria, Bangladesh, Cambodia, Dominican Republic, Egypt, India, Indonesia, Nepal, Nigeria, Pakistan, and Philippines (Table S1). While the income levels of these countries ranged from low to upper-middle, they are characterised as having moderate or good connectivity yet poor healthcare development indices. Cluster analysis of $d$ values based on the GBS and GHS indices only identified two clusters for each index, with 68 and 92 countries in the cluster closest to the WCS respectively (Table S1). Using the Rand index, Pakistan was identified as being closest to the WCS ($d$ for Rand, GBD and GHS respectively: 0.54, 0.56, 0.52), followed by India (0.55, 0.44, 0.51), Nigeria (0.61, 0.71, 0.63), Cambodia (0.63, 0.68, 0.62) and Dominican Republic (0.64, 0.71, 0.52) (Fig. 2A; Figure S4; Figure S5; Table S1).

Eleven countries were identified as being in the top five most proximal to the WCS regardless of the healthcare index used. These were India (in 3 indices’ closest five countries), Dominican Republic and Pakistan (both in 2 indices’ closest five countries), and Algeria, Cambodia, China, Indonesia, Mexico, Nigeria, Philippines and Russia (all only in 1 index’s closest five) (Table S1). Countries which were in the lowest decile proximity to the WCS across all three healthcare index derived $d$ values were Bangladesh, Dominican Republic, Egypt, India, Morocco, Pakistan and Philippines (Table S1).

Overall, there was broad agreement between the $d$ values derived from different healthcare indices (Figure S6). We found that the mean average proximity to WCS decreased with income level for Rand and GBD derived distances, but not for GHS derived distance (Fig. 2B; Figure S4B; Figure S5B). However, of the 11 countries identified above, only one (Cambodia) was classed as low income; five were lower-middle and four upper-middle income, and one (Russia) high income (Table S1).

Countries furthest away from the WCS included Slovenia (in 3 indices’ furthest five countries), Tuvalu (in 2 indices’ furthest five countries), and American Samoa, Greenland, Iceland, Luxembourg, Marshall Islands, Federated States of Micronesia, Monaco, Niue, Saint Kitts and Nevis (Leeward Islands), Swaziland, Syrian Arab Republic, Tonga and USA (all only in 1 index’s furthest five) (Table S1). The majority of these countries are classified as high income countries (with generally high healthcare indices); those that are not have poor connectivity. Some, such as Slovenia and Iceland have both high healthcare indices and poor connectivity, though we note that the potential connectivity of Iceland and Monaco may be higher were we to have included transit activity or land transportation.

## Discussion

A country with a poor ability to control and contain an outbreak of a pathogen with pandemic potential, coupled with strong connectivity to other countries, represents a notional Worst Case Scenario (WCS) as the potential source from which a new pandemic may spread globally (even if it emerged in a different location). Here, by combining information on healthcare and international travel, we have identified countries that are closest to this WCS and thereby may represent vulnerable locations which represent greatest risk in terms of initial pandemic spread. Notably, these countries were generally not those with extreme healthcare scores (either very low or very high), nor exclusively those with extreme connectivity. Our analysis indicates that the country closest to the WCS depends on the healthcare index used, but Dominican Republic, India and Pakistan featured among the closest to WCS in at least two of the index-specific analyses conducted. Other countries display similar closeness to the WCS, depending on the health care index used. These include Algeria, Cambodia, China, Indonesia, Mexico, Nigeria, Philippines and Russia. We note that two of these countries were the suspected origins of two recent pandemics following a zoonotic emergence event: China with SARS-CoV-2 coronavirus in 2019, and Mexico with AH1N1 influenza in 2009. When country scores were aggregated by income groups (as defined by the World Bank), countries in the lower-middle and upper-middle income groups tended to represent the greatest risk, whereas high income countries represented the lowest risk.

We found that global connectivity had a strong seasonal pattern, dominated by summer/winter travel in high income countries, and has increased over the time period of flight data we analysed (February 2010 to May 2015). These travel trends are in agreement with previous publications and reports^10,22,23^ indicating an annually increasing number of passengers, with seasonal variations. We also found a positive correlation between the global connectivity of a country and measures of its healthcare provision, possibly reflecting how the prosperity of a country influences both national healthcare provision and demand for travel to or from that location.

Other studies have attempted to combine measures of connectivity and healthcare provision to identify epidemic risks, though not to the extent presented here. Bogoch et al. considered the potential for international spread of plague from Madagascar through airline and the potential resulting outbreaks based on a measure of the destination’s healthcare system using the Rand index in the context of the 2017 outbreak^24^. A study of the initial dissemination of pandemic influenza A/H1N1 in 2009 through airline travel, found that incorporating healthcare factors into the model, as well as airline passenger movements, improved the model fit^16^. A further study of pandemics considered the potential number of cases generated were the pandemic to originate in different countries^25^. While this latter study did not incorporate healthcare provision in their model, their analysis identified the United States, India and China as pandemic origins with maximum impact.

Countries from where recent pandemics have originated, or present ongoing emergence events of concern, generally have good connectivity and good (though not very high) healthcare scores, according to our measures. These include: China (including Hong Kong), from where Severe Acute Respiratory Syndrome (SARS-CoV-1) emerged in 2003, and SARS-CoV-2 emerged in 2019; Mexico, from where the A/H1N1 influenza pandemic emerged in 2009; Saudi Arabia, where human cases of MERS-CoV continue to occur; Brazil, from where the Americas Zika epidemic originated in 2015. All of these countries feature in the lowest decile values (closest to WCS) of at least one of the $d$ values derived from the different healthcare indices. China was in the lowest decile $d$ value for values derived from both Rand and GBD healthcare indices. Our findings are conditional on the assumption (for each country) that they are the source of emerging pandemic. As we do not explicitly consider the country-specific probability of pandemic emergence in this analysis, addition of such a feature of countries may change the countries deemed to represent greatest risk.

Some studies have attempted to identify locations which are ‘hotspots’ for novel infectious disease emergence, often zoonotic in origin. Both our analysis and that of Jones et al.identify India, Indonesia and Bangladesh as countries causing potential problems in terms of zoonoses emergence from wildlife^26^. A holistic analysis, combining disease emergence prediction, trade and travel-based connectivity measures, and measures of epidemic preparedness would enable the identification of countries where surveillance may need to be prioritized. As the 2019 SARS-CoV-2 pandemic has shown, once a pathogen emerges from a given location, it is possible to spread internationally at great speed, particularly if adequate surveillance and communication are not prioritised or the infection is occult.

While the different healthcare indices used for our analysis generate $d$ values for countries that are broadly in agreement, there are inconsistencies. This likely reflects the different elements used and emphasis placed within each healthcare index, but also highlights the need for a widely agreed measure of ability to respond to a pandemic threat for use in this type of analysis. The scale and success of public health responses in different countries to the 2020 SARS-CoV-2 pandemic suggests that generating reliable indices is difficult.

We have identified countries whose combination of healthcare development indices and global connectivity through airline travel may indicate both a poor ability to detect and control an outbreak of a pathogen with pandemic potential and rapid potential dissemination of that pathogen to other countries. Countries that our analysis highlights as closest to the putative Worse Case Scenario country—specifically Dominican Republic, India and Pakistan, and to a lesser extent Algeria, Bangladesh, Cambodia, China, Egypt, Indonesia, Mexico, Morocco, Nigeria, Philippines and Russia—may, therefore, represent the highest risk for global health as locations for a pandemic to emerge, or a location infected in the early stages of a pandemic. They are countries with a large, often dense population, frequently living in close proximity to wildlife or livestock, with low or middle income (according to the healthcare indices we have used), and yet with moderate or good global connectivity. Improvement of these countries’ healthcare systems to detect novel outbreaks and their preparedness to control such outbreaks may be prudent^1^. We recommend an assessment of current surveillance systems, and the improvement of these and other countries’ ability to identify an outbreak of a new pathogen (through enhanced surveillance) and the countries’ ability to respond to and control such outbreaks would be beneficial not only to the origin country but also to those it is most well connected to, and ultimately all countries. Early detection of zoonotic emergence could be improved through a number of approaches, including utilising novel data streams, symptomatic and virological surveillance of community or sentinel cohorts, and the analytical integration of patient data (mortality data and incidence pattern of signature symptoms such as undiagnosed pneumonia) with livestock surveillance^27,28^. The ability of a country to control outbreaks tends to relate to logistical constraints of public health responses, such as bed capacity for the infected sick, or the effectiveness of contact tracing initiatives. We note that international development support has been established in some of these countries to help implement the World Health Organization’s International Health Regulations^29^. Our analysis may also be informative during a pandemic, as it could identify countries which could exacerbate the dissemination of a pathogen that emerged elsewhere. Thus, it could inform targeted control (such as travel bans) aimed to quench the spread of an emerged infection. Repeating this analysis at regular intervals using contemporary travel information and updated healthcare metrics may provide insights into each country’s evolving pandemic preparedness and the risk they may pose to the global community. Further work in identifying appropriate measurement of pandemic preparedness and refinement in combining such information with connectivity measures, possibly incorporating epidemic simulation, is needed.

## Limitations

There are a number of limitations to our analysis. First, the three healthcare indices used here all rely on multiple factors for their construction, of which, few were common to all and it was unclear which factors better represented a healthcare system. Additionally, we assume that all indices scaled linearly, which may not be true. Both the Rand and GBS indices considered the year 2015, and so may not reflect the current situation in some countries, though our airline data covered 2011 to 2015. Furthermore, a number of small island nations were missing from all indices (Table S1), and the indices we used also did not consider within-country administrative units, which may impact the results, especially for large countries like India and China. We note that more recent versions of the indices, specifically the GBD index, are stratified at sub-country level for large countries.

A further limitation is the use of industry-standard estimates of passenger booking numbers to derive the connectivity measure for each country. While bookings may not correspond exactly to passenger numbers, the information did include full routing (origin, destination and any layover) information. However, we did not incorporate layover transits in the country in which the connection is made in our percolation simulations. Additionally, as the booking data is proprietary, the collection and estimation methods underpinning the data are not disclosed; it is unclear the extent or size of any biases or underreporting there is in the data. As well as human movements, the speed at which a pandemic could be disseminated internationally may also depend on the pathogen’s transmission mode and ease of control^30^, and the dynamics of within-country epidemics, which we did not consider here. Models which do take these features into account have demonstrated that pathogen arrival time is a relatively simple function of the effective distance between countries afforded by airline traffic network connectivity^9^. Another consideration is that we did not consider land or sea transportation to derive our connectivity measure; we therefore likely underestimate connectivity in a number of countries. For example, the geographical dissemination of the West African Ebola outbreak 2014 to 2016 was largely driven by land transportation, rather than airline traffic. Our analysis was restricted to connectivity measures within a single year (2014) and the global number of airline passengers has since increased to 3.98 billion in 2017^31^. Integrating economic, population and travel movements is likely to provide interesting insights regarding potential changes in country proximity to WCS. Our estimate of connectivity, while related to a form of spreading (percolation) between countries, was not based on an explicit epidemic model accounting for within-country transmission or population density, which may have yielded a different ordering of countries’ connectivity. Finally, our measure of proximity to WCS assumed that our healthcare and connectivity measures are equally weighted, and implies that countries with equal distance to WCS represent identical and equal threats to global health. This is clearly a limitation of our approach, as countries as diverse as Guinea, Hungary, Trinidad and Tobago and South Korea all scored very similar distances to WCS using the Rand healthcare measure. Further work is needed to incorporate better information pertinent to a country’s ability to mitigate an epidemic, and to integrate country and pathogen specific emergence risk information^26^.

## Supplementary Information

Supplementary Information 1.

Supplementary Table S1.

## Data Availability

The airline passenger data used in this analysis during are available via subscription from OAG (http://www.oag.com); it is aggregate data containing no identifying information. Country-level income information is publicly available from The World Bank (https://datahelpdesk.worldbank.org/knowledgebase/articles/906519-world-bank-country-and-lending-groups). Country-level healthcare indices are publicly available from the cited sources for the Rand and GBD indices, and from http://www.ghsindex.org/ for the GHS index.
