# Supplementary material for: Estimating the potential for global dissemination of pandemic pathogens using the global airline network and healthcare development indices
Source: Sci Rep. 2022 Feb 23;12:3070. doi: 10.1038/s41598-022-06932-y (PMC8866520; doi:10.1038/s41598-022-06932-y)
Supplement: Supplementary file 1 — Supplementary Information 1. [file 41598_2022_6932_MOESM1_ESM.pdf]

Supplementary material

"*Estimating the potential for global dissemination of pandemic pathogens using the global airline network and healthcare development indices*"

Margaux MI Meslé, Roberto Vivancos, Ian M Hall, Robert M Christley, Steve Leach and Jonathan M Read.

Contents

Figure S1 – Temporal variation of country-level healthcare and connectivity

Figure S2 – Example percolation simulations

Figure S3 – Connectivity vs healthcare, Rand index (fully labelled)

Figure S4 – Connectivity vs healthcare, Global Burden of Disease index

Figure S5 – Connectivity vs healthcare, Global Health Security index

Figure S6 – Relationship between d values derived from different indices

Table S1 – Connectivity and healthcare metrics

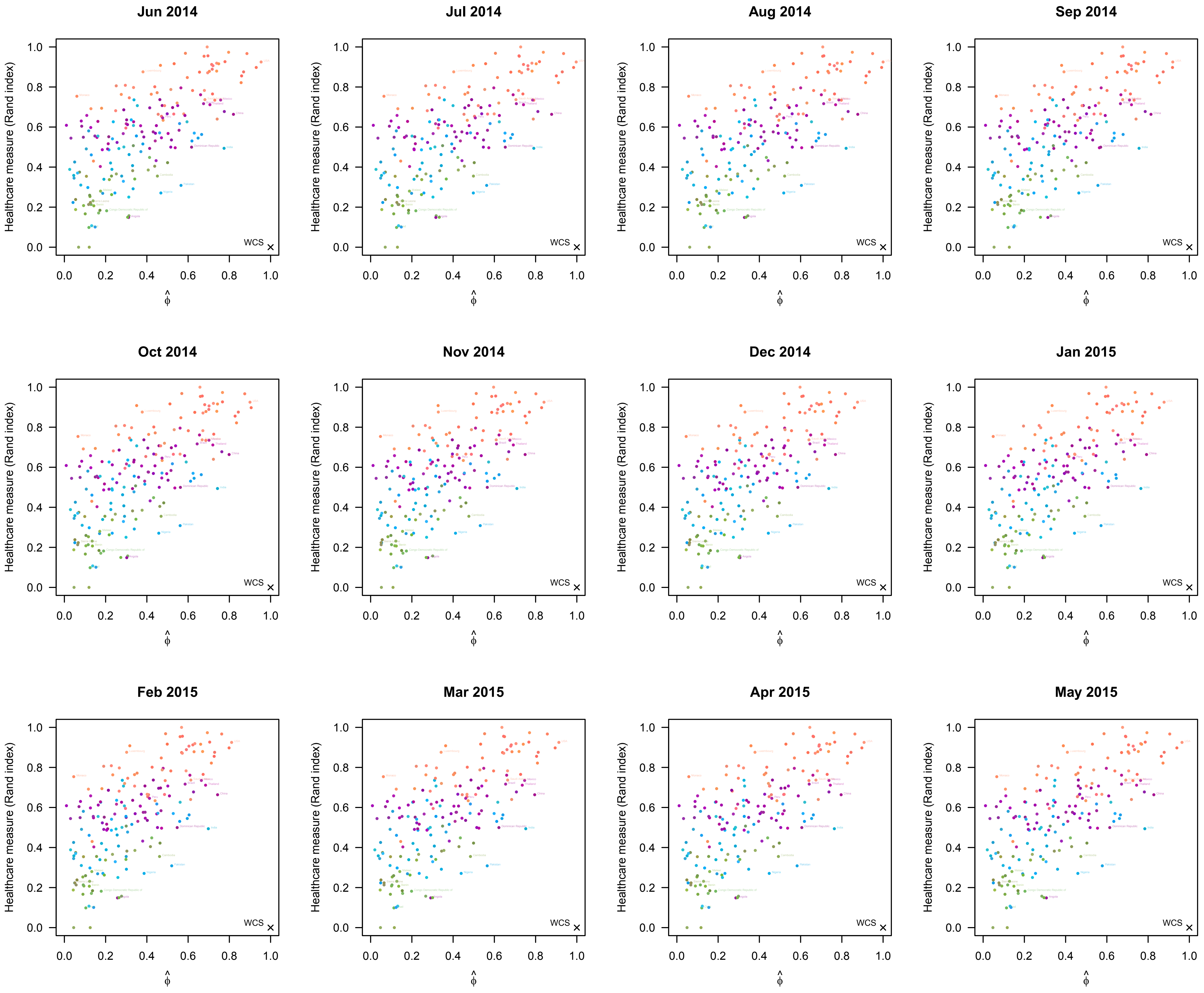

Figure S1. Temporal variation of country-level healthcare and connectivity. Country colours follow the income level coding in Figure 2. Selected countries are labelled, and the position of the Worst Case Scenario (WCS) is indicated in each panel.

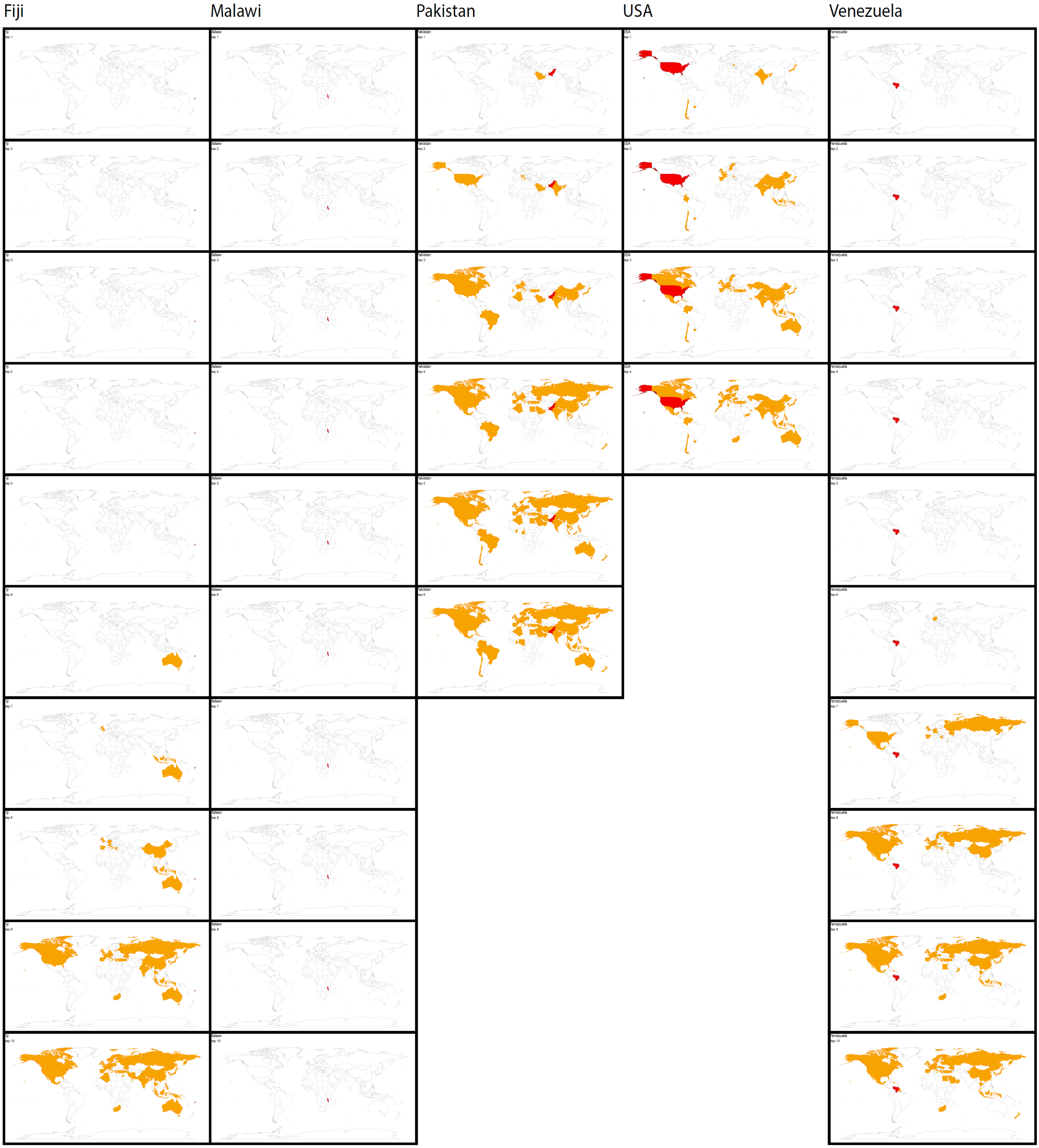

Figure S2. Example percolation simulations for selected countries, following progress for 10 percolation steps. Iterations are not shown if 25% or more of countries are reached.

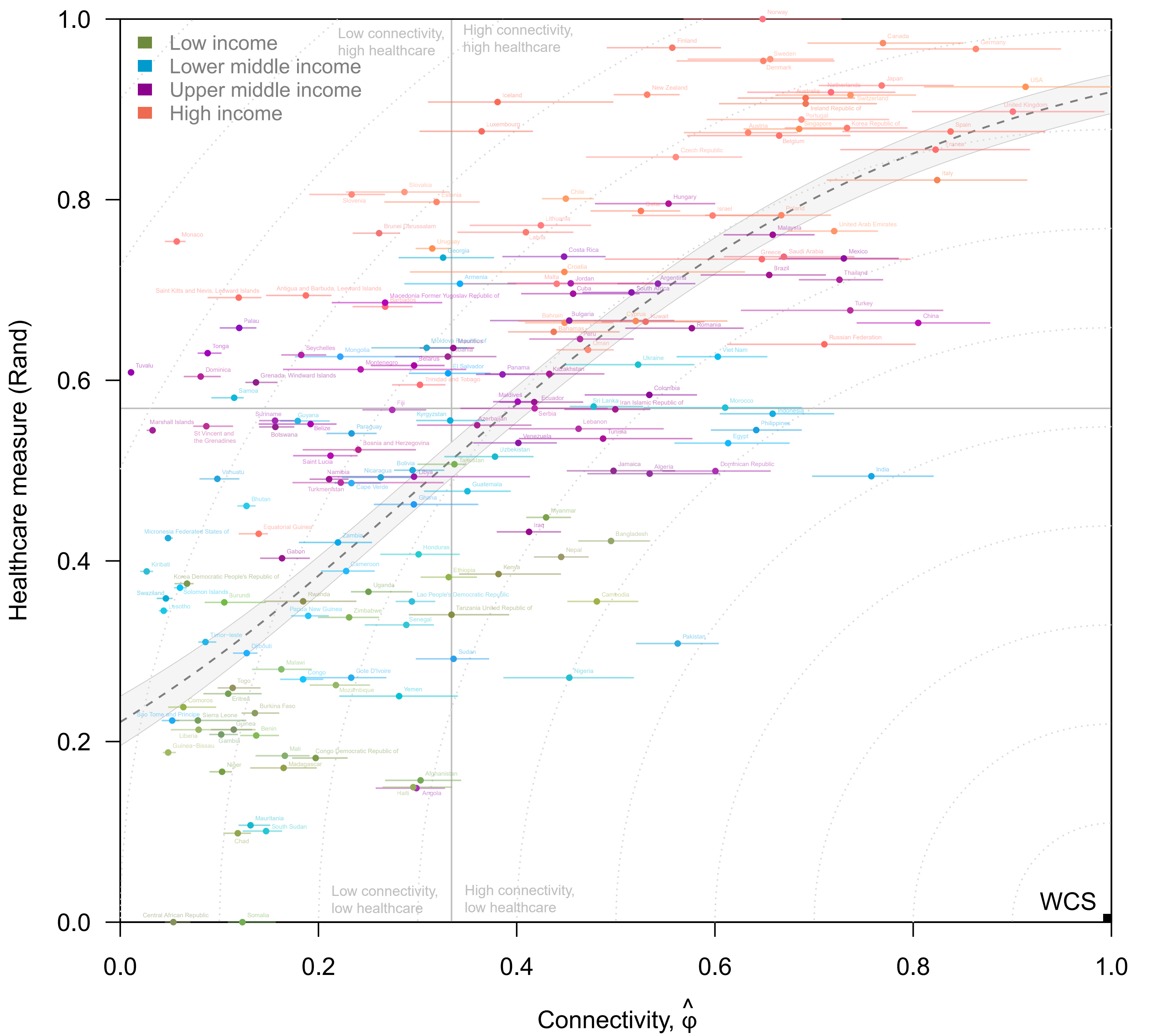

Figure S3. Relationship between healthcare measurement, Rand index, and connectivity. The average connectivity for a country is denoted by a dot, and the range by a horizontal line. A fitted spline is denoted by the dashed curve and grey area (95% confidence interval). All countries are labelled.

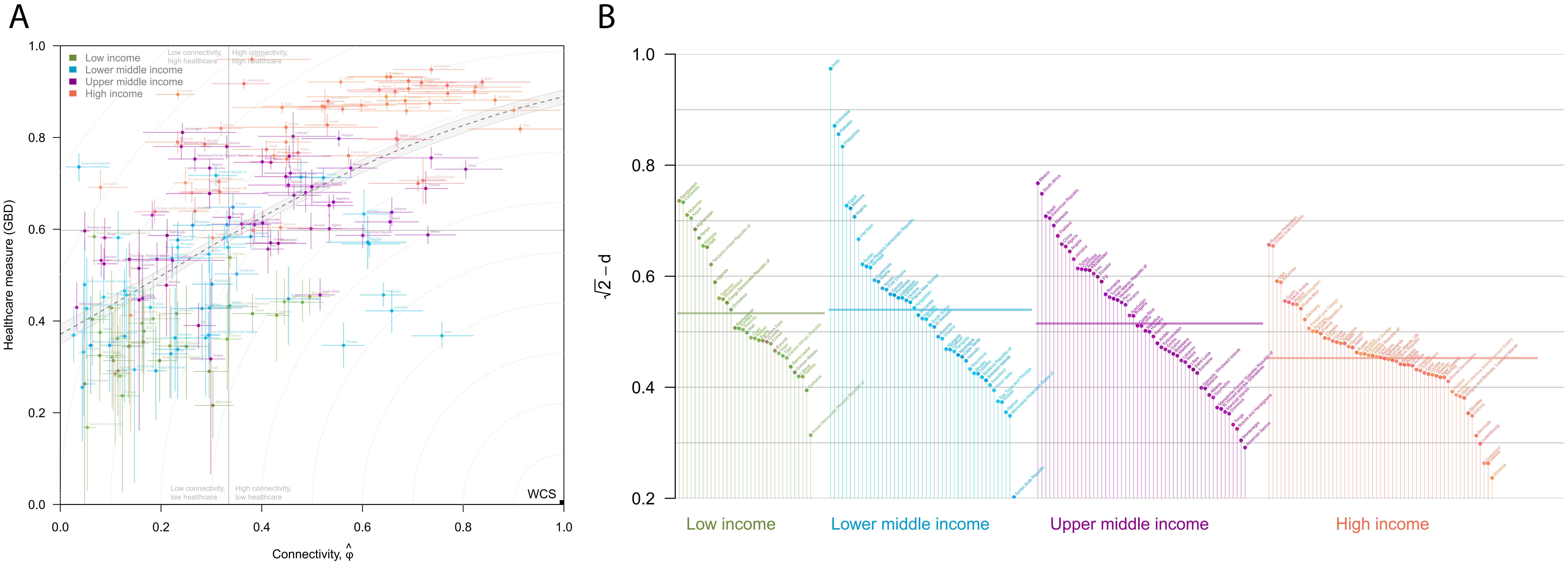

Figure S4. A, Relationship between healthcare measurement, GBD index, and connectivity. The average connectivity for a country is denoted by a dot, and the range by a horizontal line; uncertainty in the healthcare index is indicated by vertical lines for each country (95%CIs). A fitted spline is denoted by the dashed curve and grey area (95% confidence interval). B, Proximity measurement to the Worst Case Scenario (WCS) for all countries, based on the GBD healthcare index.

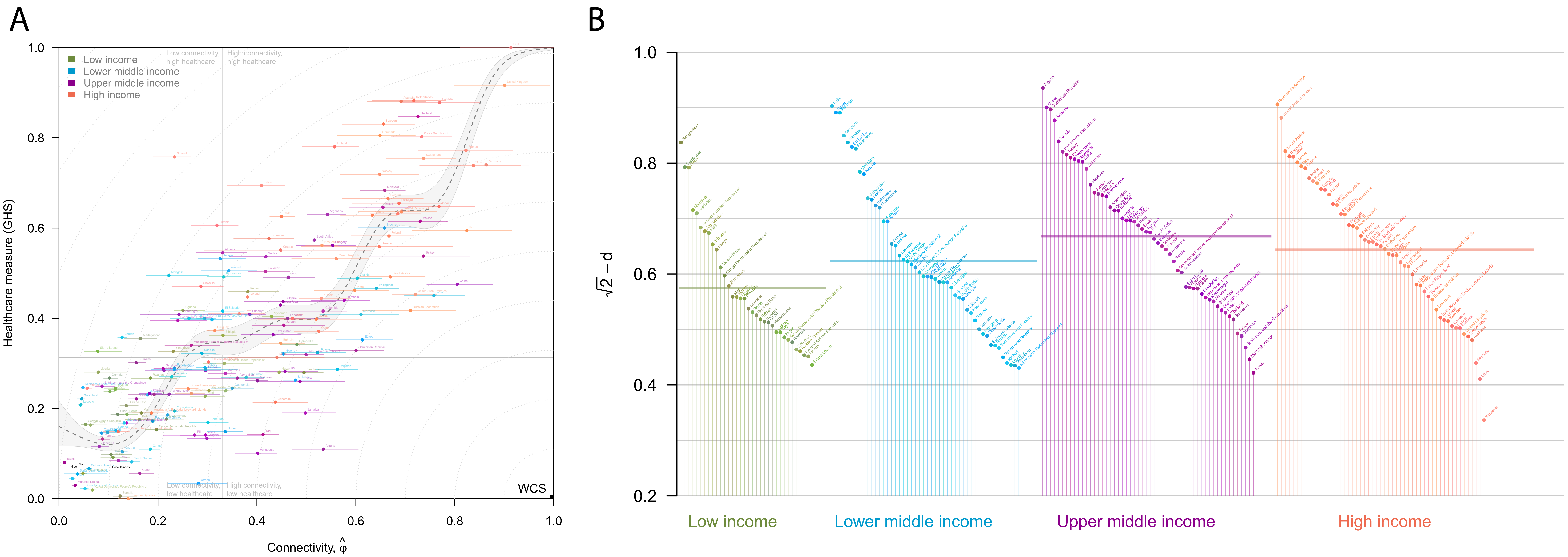

Figure S5. A, Relationship between healthcare measurement, GHS index, and connectivity. The average connectivity for a country is denoted by a dot, and the range by a horizontal line; uncertainty in the healthcare index is indicated by vertical lines for each country (95%CIs). A fitted spline is denoted by the dashed curve and grey area (95% confidence interval). B, Proximity measurement to the Worst Case Scenario (WCS) for all countries, based on the GHS healthcare index.

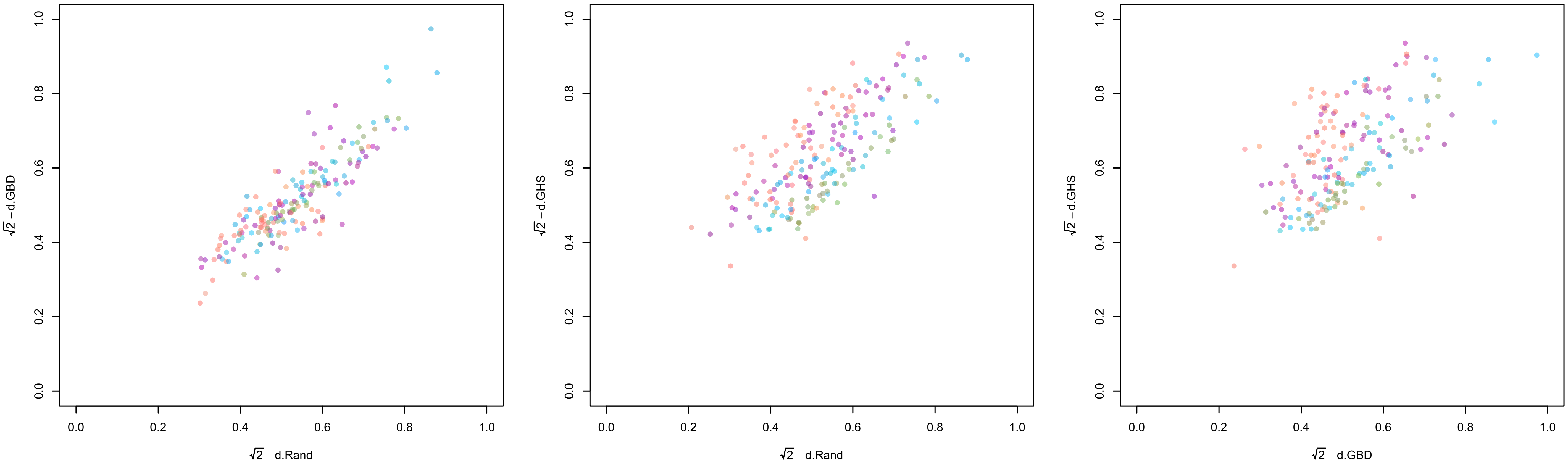

Figure S6. Pair-wise scatter plots of WCS metrics derived from Rand, GBD and GHS healthcare indices. Colours indicate income group of each country, as for Figure S3B.

Table S1. Country specific connectivity measures (ϕ), healthcare development indices (Rand, GBD, GHS), distances to the Worst Case Scenario (d) and related measures. Table rows are ordered by increasing d values, derived from the Rand index.

Country	Income group*	ϕ				Rand healthcare index					GBD healthcare index							GHS healthcare index				
		% simulations timed out	*mean*	*min*	*max*	*normalized score*	d	$\sqrt{2}-d$	*rank*	*cluster*	*normalized score*	*normalized score - 95%CI lower*	*normalized score – 95%CI upper*	d	$\sqrt{2}-d$	*rank*	*cluster*	*normalized score*	d	$\sqrt{2}-d$	*rank*	*cluster*
**Pakistan**	LM	0	0.56	0.52	0.60	0.31	0.54	0.88	1	1	0.35	0.30	0.40	0.56	0.86	3	1	0.29	0.52	0.89	7	1
**India**	LM	0	0.76	0.70	0.82	0.49	0.55	0.86	2	1	0.37	0.34	0.40	0.44	0.97	1	1	0.45	0.51	0.90	3	1
**Nigeria**	LM	0	0.45	0.39	0.52	0.27	0.61	0.80	3	1	0.45	0.35	0.52	0.71	0.71	13	1	0.32	0.63	0.78	32	1
**Cambodia**	L	0	0.48	0.45	0.52	0.36	0.63	0.79	4	1	0.44	0.40	0.49	0.68	0.73	8	1	0.34	0.62	0.79	27	1
**Dominican Republic**	UM	0	0.60	0.55	0.66	0.50	0.64	0.77	5	1	0.59	0.56	0.61	0.71	0.70	14	1	0.33	0.52	0.90	5	1
**Philippines**	LM	0	0.64	0.60	0.69	0.54	0.65	0.76	6	1	0.46	0.43	0.48	0.58	0.83	4	1	0.47	0.59	0.83	15	1
**Egypt**	LM	0	0.61	0.56	0.68	0.53	0.66	0.76	7	1	0.57	0.54	0.59	0.69	0.73	9	1	0.35	0.52	0.89	6	1
**Bangladesh**	L	0	0.50	0.46	0.53	0.42	0.66	0.76	8	1	0.45	0.41	0.49	0.68	0.74	7	1	0.28	0.58	0.84	12	1
**Indonesia**	LM	0	0.66	0.60	0.72	0.56	0.66	0.76	9	1	0.42	0.37	0.47	0.54	0.87	2	1	0.60	0.69	0.72	48	1
**Algeria**	UM	0	0.53	0.47	0.61	0.50	0.68	0.73	10	1	0.60	0.57	0.63	0.76	0.65	25	1	0.11	0.48	0.94	1	1
**Nepal**	L	0	0.44	0.42	0.47	0.40	0.69	0.73	11	1	0.44	0.39	0.49	0.71	0.70	15	1	0.28	0.62	0.79	28	1
**Morocco**	LM	0	0.61	0.53	0.69	0.57	0.69	0.72	12	2	0.57	0.51	0.63	0.69	0.72	10	1	0.41	0.56	0.85	10	1
**China**	UM	0	0.81	0.74	0.88	0.66	0.69	0.72	13	2	0.73	0.71	0.75	0.76	0.66	21	1	0.48	0.51	0.90	4	1
**Russia**	H	0	0.71	0.61	0.80	0.64	0.70	0.71	14	2	0.70	0.68	0.72	0.76	0.66	22	1	0.42	0.51	0.91	2	1
**Jamaica**	UM	0	0.50	0.45	0.56	0.50	0.71	0.71	15	2	0.60	0.56	0.64	0.78	0.63	28	1	0.19	0.54	0.88	9	1
**Afghanistan**	L	0	0.30	0.27	0.34	0.16	0.71	0.70	16	2	0.22	0.14	0.29	0.73	0.68	17	1	0.24	0.74	0.68	69	1
**Angola**	UM	0	0.30	0.26	0.33	0.15	0.72	0.70	17	2	0.32	0.07	0.49	0.77	0.64	27	1	0.13	0.71	0.70	57	1
**Haiti**	L	0	0.30	0.26	0.33	0.15	0.72	0.69	18	2	0.29	0.23	0.35	0.76	0.65	26	1	0.23	0.74	0.67	72	1
**Sudan**	LM	0	0.34	0.30	0.37	0.29	0.72	0.69	19	2	0.43	0.37	0.50	0.79	0.62	29	1	0.15	0.68	0.73	45	1
**Myanmar**	L	0	0.43	0.41	0.45	0.45	0.73	0.69	20	2	0.41	0.31	0.51	0.70	0.71	11	1	0.40	0.70	0.72	51	1
**Turkey**	UM	0	0.74	0.63	0.83	0.68	0.73	0.69	21	2	0.76	0.73	0.78	0.80	0.61	33	1	0.54	0.60	0.82	18	1
**Kenya**	L	0	0.38	0.34	0.44	0.39	0.73	0.69	22	2	0.42	0.37	0.46	0.75	0.67	19	1	0.46	0.77	0.64	87	1
**Iraq**	UM	0	0.41	0.38	0.44	0.43	0.73	0.68	23	2	0.56	0.50	0.61	0.81	0.60	37	1	0.14	0.60	0.81	21	1
**Viet Nam**	LM	0	0.60	0.56	0.65	0.63	0.74	0.67	24	2	0.63	0.58	0.69	0.75	0.67	20	1	0.49	0.63	0.78	31	1
**Tunisia**	UM	0	0.49	0.40	0.58	0.54	0.74	0.67	25	2	0.68	0.64	0.73	0.85	0.56	50	1	0.26	0.58	0.84	11	1
**Colombia**	UM	0	0.53	0.47	0.58	0.58	0.75	0.67	26	2	0.65	0.63	0.68	0.80	0.61	34	1	0.42	0.62	0.79	30	1
**Tanzania**	L	0	0.33	0.29	0.39	0.34	0.75	0.67	27	2	0.43	0.30	0.54	0.79	0.62	30	1	0.30	0.73	0.68	66	1
**Iran**	UM	0	0.50	0.45	0.53	0.57	0.76	0.66	28	3	0.69	0.65	0.73	0.85	0.56	54	1	0.32	0.59	0.82	17	1
**Yemen**	LM	0	0.28	0.22	0.34	0.25	0.76	0.65	29	3	0.43	0.31	0.53	0.84	0.58	45	1	0.03	0.72	0.69	61	1
**Thailand**	UM	0	0.73	0.68	0.77	0.71	0.76	0.65	30	3	0.69	0.65	0.73	0.74	0.67	18	1	0.85	0.89	0.52	145	2
**Lebanon**	UM	0	0.46	0.39	0.55	0.55	0.77	0.65	31	3	0.80	0.75	0.86	0.97	0.45	131	2	0.40	0.67	0.74	40	1
**Ethiopia**	L	0	0.33	0.30	0.36	0.38	0.77	0.64	32	3	0.36	0.25	0.46	0.76	0.65	24	1	0.36	0.76	0.65	80	1
**Chinese Taipei**	NA	0	0.69	0.65	0.74	0.71	0.77	0.64	33	3	NA	NA	NA	NA	NA	NA	NA	NA	NA	NA	NA	NA
**Sri Lanka**	LM	0	0.48	0.45	0.53	0.57	0.77	0.64	34	3	0.71	0.67	0.75	0.88	0.53	69	2	0.26	0.58	0.83	14	1
**Ukraine**	LM	0	0.52	0.45	0.58	0.62	0.78	0.63	35	3	0.71	0.69	0.73	0.86	0.56	57	1	0.32	0.58	0.84	13	1
**Romania**	UM	0	0.58	0.51	0.63	0.66	0.78	0.63	36	3	0.73	0.71	0.75	0.85	0.57	48	1	0.44	0.61	0.80	23	1
**Mexico**	UM	0	0.73	0.66	0.79	0.73	0.78	0.63	37	3	0.59	0.57	0.61	0.65	0.77	5	1	0.62	0.67	0.74	42	1
**Senegal**	LM	0	0.29	0.25	0.32	0.33	0.78	0.63	38	3	0.36	0.23	0.49	0.80	0.62	32	1	0.32	0.78	0.63	92	1
**Lao People's Democratic Republic**	LM	0	0.29	0.28	0.32	0.36	0.79	0.62	39	3	0.37	0.32	0.42	0.80	0.62	31	1	0.40	0.81	0.60	103	2
**Brazil**	UM	0	0.65	0.59	0.71	0.72	0.80	0.62	40	3	0.62	0.60	0.64	0.71	0.71	12	1	0.65	0.73	0.68	68	1
**Venezuela**	UM	0	0.40	0.36	0.44	0.53	0.80	0.61	41	4	0.61	0.58	0.64	0.86	0.56	56	1	0.10	0.61	0.81	22	1
**Guatemala**	LM	0	0.35	0.31	0.39	0.48	0.81	0.61	42	4	0.50	0.47	0.54	0.82	0.59	39	1	0.25	0.69	0.72	50	1
**Saudi Arabia**	H	0	0.67	0.61	0.74	0.74	0.81	0.61	43	4	0.80	0.77	0.82	0.86	0.55	59	1	0.49	0.59	0.82	16	1
**Uzbekistan**	LM	0	0.38	0.33	0.42	0.52	0.81	0.61	44	4	0.58	0.56	0.61	0.85	0.56	51	1	0.27	0.68	0.74	44	1
**Honduras**	LM	0	0.30	0.26	0.34	0.41	0.81	0.61	45	4	0.48	0.43	0.53	0.85	0.57	49	1	0.17	0.72	0.69	60	1
**Republic of Côte d'Ivoire**	LM	0	0.23	0.19	0.27	0.27	0.81	0.60	46	4	0.34	0.23	0.44	0.84	0.58	46	1	0.29	0.82	0.60	108	2
**Serbia**	UM	0	0.42	0.34	0.49	0.57	0.81	0.60	47	4	0.75	0.73	0.76	0.95	0.47	109	2	0.54	0.79	0.62	95	2
**Kuwait**	H	0	0.53	0.49	0.59	0.66	0.81	0.60	48	4	0.83	0.80	0.85	0.95	0.46	114	2	0.44	0.65	0.77	34	1
**Greece**	H	0	0.65	0.49	0.80	0.73	0.81	0.60	49	4	0.89	0.88	0.90	0.96	0.46	120	2	0.56	0.66	0.75	37	1
**United Arab Emirates**	H	0	0.72	0.67	0.76	0.77	0.81	0.60	50	4	0.71	0.65	0.76	0.76	0.65	23	1	0.45	0.53	0.88	8	1
**Ecuador**	UM	0	0.42	0.38	0.47	0.58	0.82	0.60	51	4	0.57	0.54	0.60	0.82	0.60	38	1	0.50	0.77	0.64	86	1
**Cyprus**	H	0	0.52	0.40	0.61	0.67	0.82	0.59	52	4	0.87	0.85	0.88	0.99	0.42	151	2	0.40	0.62	0.79	29	1

Country	Income group*	ϕ				Rand healthcare index					GBD healthcare index							GHS healthcare index				
		% simulations timed out	*mean*	*min*	*max*	*normalized score*	d	$\sqrt{2}-d$	*rank*	*cluster*	*normalized score*	*normalized score - 95%CI lower*	*normalized score – 95%CI upper*	d	$\sqrt{2}-d$	*rank*	*cluster*	*normalized score*	d	$\sqrt{2}-d$	*rank*	*cluster*
**Democratic Republic of Congo**	L	0	0.20	0.17	0.23	0.18	0.82	0.59	53	4	0.31	0.22	0.42	0.86	0.55	62	1	0.15	0.82	0.60	106	2
**Oman**	H	0	0.47	0.44	0.50	0.63	0.82	0.59	54	4	0.77	0.74	0.80	0.93	0.48	98	2	0.40	0.66	0.75	38	1
**Mozambique**	L	0	0.22	0.19	0.25	0.26	0.83	0.59	55	4	0.35	0.23	0.47	0.86	0.56	55	1	0.18	0.80	0.61	101	2
**Kazakhstan**	UM	0	0.43	0.37	0.49	0.61	0.83	0.58	56	4	0.57	0.55	0.60	0.80	0.61	36	1	0.36	0.67	0.74	43	1
**Maldives**	UM	0	0.40	0.38	0.42	0.58	0.83	0.58	57	4	0.75	0.72	0.78	0.96	0.46	122	2	0.26	0.65	0.76	36	1
**Uganda**	L	0	0.25	0.23	0.29	0.37	0.83	0.58	58	4	0.34	0.23	0.48	0.82	0.59	43	1	0.42	0.86	0.56	130	2
**Malaysia**	UM	0	0.66	0.61	0.70	0.76	0.83	0.58	59	4	0.64	0.61	0.67	0.72	0.69	16	1	0.68	0.76	0.65	84	1
**Tajikistan**	L	0	0.34	0.30	0.35	0.51	0.83	0.58	60	4	0.54	0.51	0.56	0.85	0.56	53	1	0.24	0.70	0.71	54	1
**Peru**	UM	0	0.46	0.41	0.52	0.65	0.84	0.58	61	4	0.67	0.64	0.70	0.86	0.55	61	1	0.49	0.73	0.69	64	1
**Zimbabwe**	L	0	0.23	0.20	0.26	0.34	0.84	0.57	62	4	0.42	0.31	0.52	0.87	0.54	68	1	0.33	0.84	0.58	116	2
**Italy**	H	0	0.82	0.71	0.92	0.82	0.84	0.57	63	4	0.91	0.90	0.92	0.93	0.49	95	2	0.59	0.62	0.79	26	1
**Argentina**	UM	0	0.54	0.50	0.58	0.71	0.84	0.57	64	4	0.66	0.64	0.68	0.80	0.61	35	1	0.63	0.78	0.64	89	1
**Ghana**	LM	0	0.30	0.26	0.36	0.46	0.84	0.57	65	4	0.43	0.31	0.54	0.82	0.59	42	1	0.29	0.76	0.65	79	1
**Azerbaijan**	UM	0	0.36	0.30	0.41	0.55	0.84	0.57	66	4	0.61	0.59	0.64	0.88	0.53	70	2	0.27	0.69	0.72	49	1
**South Africa**	UM	0	0.52	0.47	0.54	0.70	0.85	0.57	67	4	0.46	0.42	0.49	0.67	0.75	6	1	0.57	0.75	0.66	74	1
**Poland**	H	0	0.67	0.59	0.72	0.78	0.85	0.56	68	5	0.80	0.78	0.81	0.86	0.55	63	1	0.58	0.67	0.74	41	1
**Madagascar**	L	0	0.16	0.13	0.20	0.17	0.85	0.56	69	5	0.35	0.25	0.47	0.91	0.51	79	2	0.36	0.91	0.51	155	2
**Mali**	L	0	0.17	0.14	0.19	0.18	0.85	0.56	70	5	0.38	0.29	0.47	0.92	0.50	87	2	0.19	0.86	0.56	126	2
**Congo**	LM	0	0.18	0.16	0.20	0.27	0.86	0.56	71	5	NA	NA	NA	NA	NA	NA	NA	0.11	0.82	0.59	110	2
**South Sudan**	LM	0	0.15	0.12	0.16	0.10	0.86	0.56	72	5	0.29	0.05	0.47	0.90	0.51	75	2	0.08	0.86	0.56	129	2
**Libya**	UM	0	0.30	0.21	0.41	0.49	0.86	0.55	73	5	0.68	0.64	0.71	0.98	0.44	139	2	0.14	0.72	0.70	58	1
**Bulgaria**	UM	0	0.45	0.37	0.56	0.67	0.86	0.55	74	5	0.70	0.67	0.72	0.89	0.53	71	2	0.44	0.70	0.71	53	1
**Bahamas**	H	0	0.44	0.39	0.50	0.65	0.86	0.55	75	5	0.60	0.57	0.64	0.83	0.59	44	1	0.21	0.60	0.81	19	1
**Bahrain**	H	0	0.45	0.41	0.50	0.66	0.86	0.55	76	5	0.79	0.76	0.82	0.96	0.45	129	2	0.34	0.65	0.76	35	1
**Panama**	UM	0	0.39	0.36	0.42	0.61	0.86	0.55	77	5	0.61	0.57	0.64	0.87	0.55	65	1	0.41	0.74	0.68	70	1
**Cameroon**	LM	0	0.23	0.20	0.26	0.39	0.86	0.55	78	5	0.36	0.25	0.47	0.85	0.56	52	1	0.27	0.82	0.60	107	2
**Bolivia**	LM	0	0.29	0.28	0.33	0.50	0.86	0.55	79	5	0.55	0.50	0.59	0.89	0.52	73	2	0.29	0.76	0.65	82	1
**Kyrgyzstan**	LM	0	0.33	0.30	0.37	0.56	0.87	0.55	80	5	0.56	0.54	0.58	0.87	0.54	66	1	0.49	0.83	0.59	112	2
**France**	H	0	0.82	0.73	0.92	0.86	0.87	0.54	81	5	0.90	0.89	0.91	0.92	0.50	88	2	0.77	0.79	0.62	96	2
**Mauritania**	LM	0	0.13	0.12	0.15	0.11	0.88	0.54	82	5	0.46	0.36	0.56	0.98	0.43	141	2	0.17	0.88	0.53	143	2
**Somalia**	L	0	0.12	0.11	0.16	0.00	0.88	0.54	83	5	0.24	0.03	0.44	0.91	0.51	81	2	0.01	0.88	0.54	137	2
**Papua New Guinea**	LM	0	0.19	0.17	0.21	0.34	0.88	0.54	84	5	0.29	0.18	0.39	0.86	0.55	60	1	0.17	0.83	0.59	111	2
**Israel**	H	0	0.60	0.52	0.67	0.78	0.88	0.53	85	5	0.87	0.85	0.88	0.96	0.46	124	2	0.46	0.61	0.80	25	1
**Cuba**	UM	0	0.46	0.40	0.52	0.70	0.88	0.53	86	5	0.72	0.71	0.74	0.90	0.51	77	2	0.28	0.61	0.80	24	1
**Malawi**	L	0	0.16	0.13	0.19	0.28	0.88	0.53	87	5	0.40	0.29	0.50	0.93	0.49	93	2	0.18	0.86	0.56	127	2
**Zambia**	LM	0	0.22	0.18	0.25	0.42	0.89	0.53	88	5	0.33	0.23	0.43	0.85	0.57	47	1	0.19	0.80	0.61	100	2
**Nicaragua**	LM	0	0.26	0.22	0.30	0.49	0.89	0.53	89	5	0.61	0.58	0.64	0.96	0.46	119	2	0.40	0.84	0.58	118	2
**Chad**	L	0	0.12	0.10	0.13	0.10	0.89	0.53	90	5	0.28	0.15	0.41	0.92	0.49	91	2	0.19	0.90	0.51	154	2
**Benin**	L	0	0.14	0.12	0.16	0.21	0.89	0.53	91	5	0.35	0.22	0.47	0.93	0.48	96	2	0.19	0.88	0.53	141	2
**Rwanda**	L	0	0.18	0.15	0.24	0.36	0.89	0.52	92	5	0.40	0.30	0.50	0.91	0.50	82	2	0.27	0.86	0.56	131	2
**Spain**	H	0	0.84	0.73	0.93	0.88	0.89	0.52	93	5	0.92	0.91	0.93	0.94	0.48	103	2	0.74	0.76	0.66	76	1
**Jordan**	UM	0	0.45	0.39	0.54	0.71	0.89	0.52	94	5	0.76	0.73	0.78	0.93	0.48	101	2	0.38	0.67	0.75	39	1
**Burkina Faso**	L	0	0.14	0.12	0.16	0.23	0.89	0.52	95	5	0.34	0.23	0.45	0.93	0.48	97	2	0.21	0.89	0.53	144	2
**Malta**	H	0	0.44	0.34	0.54	0.71	0.90	0.51	96	6	0.87	0.85	0.88	1.03	0.38	168	2	0.31	0.64	0.77	33	1
**United Kingdom**	H	0	0.90	0.80	0.99	0.90	0.90	0.51	97	6	0.86	0.85	0.87	0.86	0.55	64	1	0.92	0.92	0.49	164	2
**El Salvador**	LM	0	0.33	0.29	0.37	0.61	0.90	0.51	98	6	0.61	0.57	0.64	0.91	0.51	78	2	0.42	0.79	0.63	93	2
**Croatia**	H	0	0.45	0.29	0.63	0.72	0.91	0.51	99	6	0.82	0.81	0.84	0.99	0.42	150	2	0.55	0.78	0.63	90	1
**Cape Verde**	LM	0	0.23	0.22	0.26	0.49	0.91	0.51	100	6	0.58	0.53	0.62	0.96	0.45	125	2	0.19	0.79	0.62	94	2
**Guinea**	L	0	0.11	0.09	0.13	0.21	0.91	0.50	101	6	0.29	0.19	0.39	0.93	0.48	99	2	0.25	0.92	0.50	160	2
**Hungary**	UM	0	0.55	0.48	0.60	0.80	0.91	0.50	102	6	0.80	0.78	0.81	0.91	0.50	86	2	0.56	0.72	0.70	59	1
**Niger**	L	0	0.10	0.09	0.11	0.17	0.91	0.50	103	6	0.32	0.21	0.42	0.95	0.46	115	2	0.24	0.93	0.49	168	2
**Albania**	UM	0	0.33	0.28	0.38	0.63	0.92	0.50	104	6	0.78	0.75	0.80	1.03	0.39	167	2	0.55	0.86	0.55	135	2
**Trinidad and Tobago**	H	0	0.30	0.27	0.33	0.59	0.92	0.50	105	6	0.58	0.55	0.61	0.91	0.51	80	2	0.30	0.76	0.65	81	1
**Turkmenistan**	UM	0	0.22	0.17	0.33	0.49	0.92	0.50	106	6	0.53	0.51	0.56	0.94	0.47	106	2	0.23	0.81	0.60	104	2
**South Korea**	H	0	0.73	0.67	0.79	0.88	0.92	0.50	107	6	0.87	0.86	0.89	0.91	0.50	85	2	0.80	0.85	0.57	122	2
**Mauritius**	UM	0	0.34	0.30	0.36	0.64	0.92	0.49	108	6	0.63	0.60	0.65	0.91	0.50	83	2	0.28	0.72	0.69	62	1
**Qatar**	H	0	0.53	0.47	0.57	0.79	0.92	0.49	109	6	0.87	0.83	0.90	0.99	0.43	146	2	0.37	0.60	0.81	20	1
**Costa Rica**	UM	0	0.45	0.39	0.49	0.74	0.92	0.49	110	6	0.71	0.69	0.74	0.90	0.51	76	2	0.43	0.70	0.71	52	1

Country	Income group*	ϕ				Rand healthcare index					GBD healthcare index							GHS healthcare index				
		% simulations timed out	*mean*	*min*	*max*	*normalized score*	d	$\sqrt{2}-d$	*rank*	*cluster*	*normalized score*	*normalized score - 95%CI lower*	*normalized score – 95%CI upper*	d	$\sqrt{2}-d$	*rank*	*cluster*	*normalized score*	d	$\sqrt{2}-d$	*rank*	*cluster*
**Fiji**	UM	0	0.27	0.24	0.31	0.57	0.92	0.49	111	6	0.39	0.34	0.44	0.82	0.59	41	1	0.14	0.74	0.68	71	1
**Gambia**	L	0	0.10	0.09	0.12	0.21	0.92	0.49	112	6	0.43	0.35	0.51	1.00	0.42	154	2	0.27	0.94	0.48	171	2
**Djibouti**	LM	0	0.13	0.11	0.14	0.30	0.92	0.49	113	6	0.37	0.22	0.49	0.95	0.47	110	2	0.10	0.88	0.54	138	2
**Bosnia and Herzegovina**	UM	0	0.24	0.18	0.30	0.52	0.92	0.49	114	6	0.78	0.75	0.80	1.09	0.33	182	2	0.40	0.86	0.56	128	2
**Togo**	L	0	0.11	0.10	0.14	0.26	0.92	0.49	115	6	0.36	0.27	0.46	0.96	0.46	123	2	0.24	0.92	0.50	161	2
**Eritrea**	L	0	0.11	0.08	0.14	0.25	0.93	0.49	116	6	0.29	0.13	0.43	0.94	0.48	104	2	0.09	0.90	0.52	147	2
**Gabon**	UM	0	0.16	0.14	0.19	0.40	0.93	0.49	117	6	0.45	0.34	0.54	0.95	0.46	113	2	0.06	0.84	0.58	119	2
**USA**	H	0	0.91	0.81	1.00	0.92	0.93	0.49	118	6	0.82	0.81	0.83	0.82	0.59	40	1	1.00	1.00	0.41	191	2
**Namibia**	UM	0	0.21	0.19	0.23	0.49	0.93	0.48	119	6	0.48	0.37	0.57	0.92	0.49	89	2	0.29	0.84	0.57	120	2
**Singapore**	H	0	0.68	0.63	0.73	0.88	0.93	0.48	120	6	0.88	0.87	0.89	0.93	0.48	102	2	0.63	0.71	0.71	55	1
**Belgium**	H	0	0.66	0.57	0.74	0.87	0.93	0.48	121	6	0.90	0.89	0.91	0.96	0.45	126	2	0.67	0.75	0.67	73	1
**Belarus**	UM	0	0.30	0.25	0.33	0.62	0.94	0.48	122	6	0.73	0.71	0.75	1.02	0.40	162	2	0.28	0.76	0.66	78	1
**Paraguay**	LM	0	0.23	0.21	0.26	0.54	0.94	0.48	123	6	0.56	0.52	0.60	0.95	0.46	112	2	0.29	0.82	0.59	109	2
**Republic of Moldova**	LM	0	0.31	0.25	0.35	0.64	0.94	0.48	124	6	0.72	0.69	0.74	1.00	0.42	155	2	0.40	0.80	0.62	97	2
**Saint Lucia**	UM	0	0.21	0.17	0.24	0.52	0.94	0.47	125	7	0.59	0.55	0.62	0.98	0.43	142	2	0.28	0.84	0.58	117	2
**Portugal**	H	0	0.69	0.59	0.78	0.89	0.94	0.47	126	7	0.86	0.85	0.87	0.91	0.50	84	2	0.66	0.73	0.69	63	1
**Liberia**	L	0	0.08	0.05	0.14	0.21	0.95	0.47	127	7	0.38	0.28	0.47	0.99	0.42	153	2	0.28	0.96	0.45	179	2
**Central African Republic**	L	0.1	0.05	0.05	0.07	0.00	0.95	0.47	128	7	0.17	0.03	0.32	0.96	0.45	127	2	0.16	0.96	0.45	178	2
**Austria**	H	0	0.63	0.57	0.68	0.87	0.95	0.47	129	7	0.90	0.89	0.91	0.98	0.44	138	2	0.63	0.73	0.69	65	1
**Sierra Leone**	L	0	0.08	0.05	0.13	0.22	0.95	0.47	130	7	0.32	0.23	0.42	0.98	0.44	140	2	0.33	0.98	0.44	185	2
**Switzerland**	H	0	0.74	0.66	0.80	0.92	0.95	0.46	131	7	0.95	0.94	0.96	0.98	0.43	144	2	0.75	0.80	0.61	98	2
**Czech Republic**	H	0	0.56	0.47	0.63	0.85	0.95	0.46	132	7	0.86	0.85	0.87	0.97	0.45	133	2	0.53	0.69	0.72	47	1
**Japan**	H	0	0.77	0.70	0.84	0.93	0.95	0.46	133	7	0.91	0.90	0.92	0.94	0.47	107	2	0.65	0.69	0.73	46	1
**Republic of Ireland**	H	0	0.69	0.60	0.76	0.91	0.96	0.46	134	7	0.91	0.90	0.92	0.96	0.46	121	2	0.64	0.71	0.71	56	1
**Netherlands**	H	0	0.72	0.63	0.78	0.92	0.96	0.45	135	7	0.92	0.91	0.93	0.96	0.45	128	2	0.88	0.93	0.49	167	2
**Equatorial Guinea**	H	0	0.14	0.12	0.15	0.43	0.96	0.45	136	7	0.41	0.16	0.55	0.95	0.46	118	2	0.00	0.86	0.55	133	2
**Burundi**	L	0	0.11	0.09	0.15	0.35	0.96	0.45	137	7	0.31	0.20	0.42	0.95	0.47	111	2	0.10	0.90	0.51	153	2
**Lithuania**	H	0	0.42	0.35	0.48	0.77	0.96	0.45	138	7	0.76	0.75	0.78	0.95	0.46	116	2	0.58	0.81	0.60	105	2
**Australia**	H	0	0.69	0.62	0.74	0.91	0.96	0.45	139	7	0.92	0.91	0.93	0.97	0.44	136	2	0.88	0.93	0.48	170	2
**Timor-Leste**	LM	0	0.09	0.08	0.10	0.31	0.97	0.45	140	7	0.45	0.39	0.52	1.02	0.39	163	2	0.15	0.93	0.49	165	2
**Armenia**	LM	0	0.34	0.29	0.40	0.71	0.97	0.45	141	7	0.65	0.62	0.68	0.92	0.49	90	2	0.51	0.83	0.59	113	2
**Latvia**	H	0	0.41	0.34	0.46	0.76	0.97	0.45	142	7	0.77	0.76	0.79	0.97	0.44	137	2	0.69	0.91	0.50	157	2
**Comoros**	L	0	0.06	0.05	0.10	0.24	0.97	0.45	143	7	0.40	0.30	0.50	1.02	0.39	164	2	0.16	0.95	0.46	176	2
**Guinea-Bissau**	L	0	0.05	0.04	0.06	0.19	0.97	0.44	144	7	0.26	0.00	0.43	0.99	0.43	145	2	0.06	0.95	0.46	177	2
**Chile**	H	0	0.45	0.43	0.48	0.80	0.97	0.44	145	7	0.75	0.73	0.77	0.93	0.48	100	2	0.63	0.83	0.58	114	2
**São Tomé and Príncipe**	LM	0	0.05	0.04	0.06	0.22	0.97	0.44	146	7	0.43	0.32	0.54	1.04	0.37	171	2	0.02	0.95	0.47	175	2
**Montenegro**	UM	0	0.24	0.16	0.35	0.61	0.97	0.44	147	7	0.81	0.79	0.83	1.11	0.30	185	2	0.41	0.86	0.55	134	2
**Germany**	H	0	0.86	0.76	0.95	0.97	0.98	0.44	148	7	0.88	0.87	0.89	0.89	0.52	74	2	0.74	0.75	0.66	75	1
**Belize**	UM	0	0.19	0.14	0.22	0.55	0.98	0.44	149	7	0.53	0.50	0.57	0.97	0.45	134	2	0.23	0.84	0.57	121	2
**Bhutan**	LM	0	0.13	0.12	0.14	0.46	0.99	0.43	150	8	0.47	0.41	0.51	0.99	0.43	148	2	0.36	0.94	0.47	173	2
**Guyana**	LM	0	0.18	0.16	0.21	0.55	0.99	0.42	151	8	0.43	0.39	0.47	0.93	0.49	94	2	0.23	0.85	0.56	124	2
**Georgia**	LM	0	0.33	0.28	0.38	0.74	1.00	0.42	152	8	0.58	0.56	0.61	0.89	0.52	72	2	0.53	0.86	0.56	132	2
**Mongolia**	LM	0	0.22	0.18	0.30	0.63	1.00	0.42	153	8	0.54	0.49	0.57	0.95	0.47	108	2	0.49	0.92	0.49	163	2
**Canada**	H	0	0.77	0.69	0.85	0.97	1.00	0.41	154	8	0.90	0.89	0.91	0.93	0.49	92	2	0.88	0.91	0.51	156	2
**Barbados**	H	0	0.27	0.23	0.29	0.68	1.00	0.41	155	8	0.64	0.61	0.67	0.97	0.44	135	2	0.23	0.77	0.65	85	1
**Macedonia**	UM	0	0.27	0.21	0.32	0.69	1.00	0.41	156	8	0.75	0.73	0.78	1.05	0.36	173	2	0.34	0.81	0.61	102	2
**North Korea**	L	0	0.07	0.05	0.07	0.37	1.01	0.41	157	8	0.58	0.52	0.64	1.10	0.31	183	2	0.02	0.93	0.48	169	2
**Botswana**	UM	0	0.16	0.14	0.18	0.55	1.01	0.41	158	8	0.45	0.16	0.60	0.95	0.46	117	2	0.22	0.87	0.54	136	2
**Solomon Islands**	LM	0	0.06	0.05	0.07	0.37	1.01	0.40	159	8	0.35	0.24	0.45	1.00	0.41	158	2	0.07	0.94	0.47	172	2
**Suriname**	UM	0	0.16	0.14	0.18	0.56	1.01	0.40	160	8	0.51	0.48	0.55	0.99	0.43	147	2	0.30	0.90	0.52	148	2
**Uruguay**	H	0	0.31	0.30	0.33	0.75	1.01	0.40	161	8	0.70	0.69	0.72	0.98	0.43	143	2	0.37	0.78	0.63	91	1
**Sweden**	H	0	0.66	0.57	0.72	0.96	1.02	0.40	162	8	0.93	0.92	0.94	0.99	0.42	152	2	0.83	0.90	0.52	151	2
**Denmark**	H	0	0.65	0.56	0.72	0.95	1.02	0.40	163	8	0.87	0.86	0.89	0.94	0.47	105	2	0.81	0.88	0.54	139	2
**Lesotho**	LM	0	0.04	0.04	0.05	0.34	1.02	0.40	164	8	0.26	0.14	0.38	0.99	0.42	149	2	0.21	0.98	0.44	186	2
**Swaziland**	LM	0.4	0.05	0.04	0.05	0.36	1.02	0.40	165	8	0.33	0.19	0.49	1.01	0.40	160	2	0.22	0.98	0.43	187	2
**Vanuatu**	LM	0	0.10	0.08	0.12	0.49	1.03	0.39	166	8	0.35	0.25	0.43	0.97	0.45	132	2	0.15	0.91	0.50	159	2
**New Zealand**	H	0	0.53	0.50	0.56	0.92	1.03	0.39	167	8	0.88	0.87	0.89	1.00	0.42	156	2	0.56	0.73	0.68	67	1
**Seychelles**	UM	0	0.18	0.16	0.21	0.63	1.03	0.38	168	8	0.63	0.60	0.66	1.03	0.38	169	2	0.23	0.85	0.56	123	2

Country	Income group*	ϕ				Rand healthcare index					GBD healthcare index							GHS healthcare index				
		% simulations timed out	*mean*	*min*	*max*	*normalized score*	d	$\sqrt{2}-d$	*rank*	*cluster*	*normalized score*	*normalized score - 95%CI lower*	*normalized score – 95%CI upper*	d	$\sqrt{2}-d$	*rank*	*cluster*	*normalized score*	d	$\sqrt{2}-d$	*rank*	*cluster*
**Federated States of Micronesia**	LM	0.1	0.05	0.04	0.05	0.43	1.04	0.37	169	9	0.48	0.36	0.57	1.07	0.35	179	2	0.25	0.98	0.43	188	2
**Kiribati**	LM	3.3	0.03	0.02	0.03	0.39	1.05	0.37	170	9	0.37	0.31	0.43	1.04	0.37	172	2	0.04	0.97	0.44	184	2
**Estonia**	H	0	0.32	0.27	0.36	0.80	1.05	0.37	171	9	0.82	0.80	0.83	1.07	0.35	180	2	0.61	0.91	0.50	158	2
**Grenada, Windward Islands**	UM	0	0.14	0.13	0.16	0.60	1.05	0.36	172	9	0.53	0.50	0.57	1.02	0.40	161	2	0.17	0.88	0.53	140	2
**Samoa**	LM	0	0.11	0.09	0.12	0.58	1.06	0.36	173	9	0.58	0.53	0.64	1.06	0.36	176	2	0.15	0.90	0.52	150	2
**Norway**	H	0	0.65	0.57	0.73	1.00	1.06	0.35	174	9	0.93	0.92	0.94	1.00	0.42	157	2	0.72	0.80	0.61	99	2
**Brunei**	H	0	0.26	0.23	0.28	0.76	1.06	0.35	175	9	0.68	0.66	0.70	1.00	0.41	159	2	0.24	0.78	0.64	88	1
**Finland**	H	0	0.56	0.49	0.61	0.97	1.06	0.35	176	9	0.92	0.91	0.93	1.02	0.39	165	2	0.78	0.90	0.52	149	2
**St Vincent and the Grenadines**	UM	0	0.09	0.07	0.11	0.55	1.07	0.35	177	9	0.52	0.49	0.56	1.05	0.36	174	2	0.25	0.95	0.47	174	2
**Antigua and Barbuda, Leeward Islands**	H	0	0.19	0.15	0.21	0.69	1.07	0.35	178	9	0.64	0.61	0.67	1.03	0.38	170	2	0.19	0.83	0.58	115	2
**Slovakia**	H	0	0.29	0.23	0.33	0.81	1.08	0.34	179	9	0.79	0.77	0.80	1.06	0.35	177	2	0.47	0.85	0.56	125	2
**Luxembourg**	H	0	0.36	0.30	0.42	0.88	1.08	0.33	180	9	0.92	0.91	0.93	1.12	0.30	186	2	0.41	0.76	0.66	77	1
**Palau**	UM	0	0.12	0.10	0.14	0.66	1.10	0.32	181	10	NA	NA	NA	NA	NA	NA	NA	0.08	0.88	0.53	142	2
**Iceland**	H	0	0.38	0.31	0.50	0.91	1.10	0.32	182	10	0.97	0.96	0.98	1.15	0.26	189	2	0.45	0.76	0.65	83	1
**Dominica**	UM	0	0.08	0.06	0.10	0.60	1.10	0.31	183	10	0.53	0.50	0.57	1.06	0.35	178	2	0.12	0.93	0.49	166	2
**Tonga**	UM	0	0.09	0.08	0.10	0.63	1.11	0.31	184	10	0.58	0.53	0.62	1.08	0.33	181	2	0.13	0.92	0.49	162	2
**Marshall Islands**	UM	0.9	0.03	0.03	0.04	0.54	1.11	0.30	185	10	0.43	0.38	0.48	1.06	0.36	175	2	0.03	0.97	0.45	182	2
**Slovenia**	H	0	0.23	0.19	0.27	0.81	1.11	0.30	186	10	0.89	0.88	0.91	1.18	0.24	190	2	0.76	1.08	0.34	192	2
**Saint Kitts and Nevis, Leeward Islands**	H	0	0.12	0.09	0.14	0.69	1.12	0.29	187	10	NA	NA	NA	NA	NA	NA	NA	0.15	0.89	0.52	146	2
**Tuvalu**	UM	30.3	0.01	0.01	0.01	0.61	1.16	0.25	188	10	NA	NA	NA	NA	NA	NA	NA	0.08	0.99	0.42	190	2
**Monaco**	H	0	0.06	0.05	0.07	0.75	1.21	0.21	189	10	NA	NA	NA	NA	NA	NA	NA	0.25	0.97	0.44	183	2
**American Samoa**	UM	0	0.05	0.04	0.06	NA	NA	NA	NA	NA	0.60	0.55	0.64	1.12	0.29	187	2	NA	NA	NA	NA	NA
**Andorra**	H	NA	NA	NA	NA	NA	NA	NA	NA	NA	0.98	0.97	1.00	NA	NA	NA	NA	0.21	NA	NA	NA	NA
**Bermuda**	H	0	0.23	0.17	0.28	NA	NA	NA	NA	NA	0.79	0.77	0.81	1.10	0.31	184	2	NA	NA	NA	NA	NA
**Congo (Brazzaville)**	NA	NA	NA	NA	NA	NA	NA	NA	NA	NA	0.35	0.24	0.47	NA	NA	NA	NA	NA	NA	NA	NA	NA
**Cook Islands**	NA	0	0.10	0.07	0.13	NA	NA	NA	NA	NA	NA	NA	NA	NA	NA	NA	NA	0.06	0.90	0.51	152	2
**Greenland**	H	0	0.08	0.05	0.13	NA	NA	NA	NA	NA	0.69	0.66	0.73	1.15	0.26	188	2	NA	NA	NA	NA	NA
**Guam**	H	0	0.38	0.37	0.40	NA	NA	NA	NA	NA	0.60	0.56	0.64	0.86	0.56	58	1	NA	NA	NA	NA	NA
**Liechtenstein**	H	100	NA	NA	NA	NA	NA	NA	NA	NA	NA	NA	NA	NA	NA	NA	NA	0.41	NA	NA	NA	NA
**Nauru**	NA	0.4	0.04	0.03	0.04	NA	NA	NA	NA	NA	NA	NA	NA	NA	NA	NA	NA	0.07	0.97	0.45	181	2
**Niue**	NA	5.7	0.02	0.01	0.03	NA	NA	NA	NA	NA	NA	NA	NA	NA	NA	NA	NA	0.06	0.98	0.43	189	2
**Northern Mariana Islands (except Guam)**	H	0	0.25	0.22	0.26	NA	NA	NA	NA	NA	0.70	0.66	0.74	1.03	0.39	166	2	NA	NA	NA	NA	NA
**Palestine**	NA	NA	NA	NA	NA	NA	NA	NA	NA	NA	0.69	0.64	0.73	NA	NA	NA	NA	NA	NA	NA	NA	NA
**Puerto Rico**	H	0	0.57	0.52	0.64	NA	NA	NA	NA	NA	0.76	0.74	0.78	0.87	0.54	67	1	NA	NA	NA	NA	NA
**San Marino**	H	100	NA	NA	NA	NA	NA	NA	NA	NA	NA	NA	NA	NA	NA	NA	NA	0.22	NA	NA	NA	NA
**Syria**	LM	11.4	0.04	0.01	0.10	NA	NA	NA	NA	NA	0.74	0.70	0.77	1.21	0.20	191	2	0.05	0.96	0.45	180	2
**Taiwan SAR, China**	H	NA	NA	NA	NA	NA	NA	NA	NA	NA	0.77	0.74	0.81	NA	NA	NA	NA	NA	NA	NA	NA	NA
**Virgin Islands, USA**	H	0	0.32	0.24	0.36	NA	NA	NA	NA	NA	0.68	0.65	0.71	0.97	0.45	130	2	NA	NA	NA	NA	NA

* income group: L = low; LM = lower-middle; UM = upper middle; H = high.
